# Unilateral Hydronephrosis and Renal Damage after Acute Leukemia

**DOI:** 10.1155/2012/968491

**Published:** 2012-04-03

**Authors:** Egle Simanauskiene, Valentina Daugelaviciene, Arvydas Laurinavicius, Ugnius Mickys, Vaida Simonyte, Goda Vaitkeviciene, Gilvydas Verkauskas

**Affiliations:** ^1^Children's Hospital, Vilnius University Hospital Santariskiu Klinikos, Santariskiu Street 7, 08406 Vilnius, Lithuania; ^2^National Center of Pathology, Vilnius University Hospital Santariskiu Klinikos, Santariskiu Street 2, 08661 Vilnius, Lithuania

## Abstract

A 14-year-old boy presented with asymptomatic right hydronephrosis detected on routine yearly ultrasound examination. Previously, he had at least two normal renal ultrasonograms, 4 years after remission of acute myeloblastic leukemia, treated by AML-BFM-93 protocol. A function of the right kidney and no damage on the left was confirmed by a DMSA scan. Right retroperitoneoscopic nephrectomy revealed 3 renal arteries with the lower pole artery lying on the pelviureteric junction. Histologically chronic tubulointerstitial nephritis was detected. In the pathogenesis of this severe unilateral renal damage, we suspect the exacerbation of deleterious effects of cytostatic therapy on kidneys with intermittent hydronephrosis.

## 1. Introduction

Renal damage as a consequence of hematologic malignancy and cytostatic therapy is well known; however, we report an unusual case of unilateral kidney lesion associated with obstructive uropathy. Obstruction to urinary flow can sometimes be difficult to diagnose. It was probably best defined as “any restriction to urinary outflow that left untreated will cause progressive renal deterioration” [1]. Relation between the degree and the duration of obstruction to the amount of damage is still not evident, similarly as renal capacity to recover after elimination of obstruction. Generally, symptoms can warn the clinicians about deleterious effects of obstruction. But asymptomatic damage is also well known, subjecting patients with a dilatation of renal collecting system to numerous exams. It remains unclear whether cytostatic therapy or hematologic malignancy could potentiate renal damage of hydronephrotic kidney.

## 2. Case Report

A 14-year-old boy presented with right hydronephrosis. He had no complaints, and this was an unexpected finding detected by ultrasonography during his periodical health test. The boy had a past history of acute myeloblastic leukemia at 8 years of age. The disease was treated according to AML-BFM-93 protocol, which includes cytarabine, vepezide, daunorubicine, prednisolone, vincristine, adriomycin, cyclophosphamide, and tioguanine. A complete remission was achieved 4 years ago. No signs of renal lesion were recorded during and after chemotherapy. Previous yearly ultrasound examinations showed no alteration of the kidneys also. Having detected hydronephrosis, the patient was admitted for further examination and treatment to the Department of Urology.

Ultrasonography showed hydronephrosis on the right with anteroposterior diameter of pelvis reaching 50 mm, calyceal dilatation and parenchymal thinning. Ureter was not dilated. The left kidney seemed normal. Calculus obstructing the pyeloureteral segment was suspected on the first ultrasound test. However, no sign of urinary stone was detected on Doppler ultrasound, confirming accessory vessel extrinsic to pyeloureteral junction.

His general blood test and urinalysis were normal, and the serum concentration of creatinine was 59 *μ*mol/L. Intravenous urography revealed normal left kidney, and no excretion of the contrast material was seen on the right side even on the late urograms. 99Tc-dimercaptosuccinic acid (DMSA) scan was made to assess differential renal function. It showed no renal uptake of DMSA on the right. No anatomical or functional lesion was found in the left kidney.

**Figure 1:**
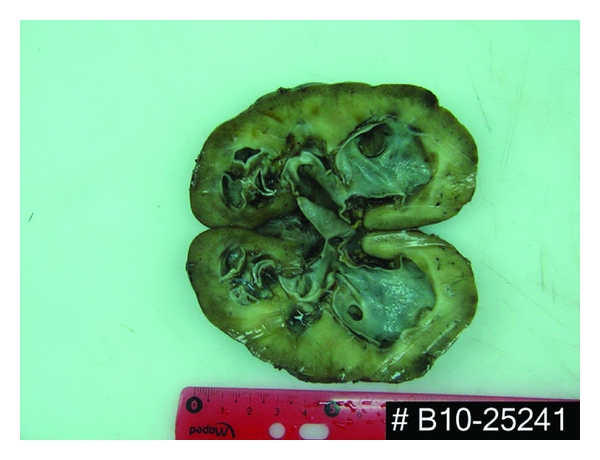
Macroscopic view of the kidney.

**Figure 2:**
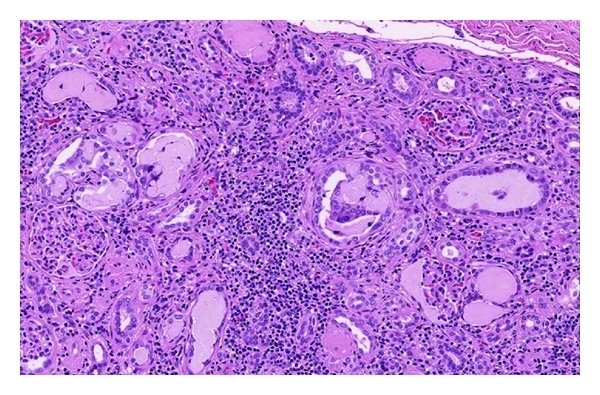
Histopathological view.

Nephrostomy was proposed as initial management with a little hope for some part of renal function to recover. The boy and his parents refused, willing for a definite procedure. The right retroperitoneoscopic nephrectomy was performed. Three renal arteries were found with the lower pole artery lying on pyeloureteral junction. The kidney was 10.5 × 5.5 cm of size, with slight atrophy of the parenchyma (Figure 1). There were no intraoperative or postoperative complications. Histopathology revealed chronic tubulointerstitial nephritis. Glomeruli showed no significant changes. A rather copious infiltration of lymphocytes, several plasmocytes, and granulocytes were present in stroma. An obstruction of tubules with a prominent amount of small eosinophilic and periodic acid-Schiff (PAS) positive cylinders was found. Tubular epithelium was focally detached. There was a peritubular fibrosis around excretory tubules (Figure 2). Special attention was focused on the search of BK nephritis, but no specific histological signs were found.

## 3. Discussion

The case is intriguing because of unusual combination. Unilateral damage is difficult to explain by the toxic effects of chemotherapy. No function on DMSA scan and intravenous urography do not correlate with macroscopic findings of removed kidney and no previous history of hydronephrosis.

Histological investigation also did not provide a straightforward answer. There was no widespread glomerular collapse and tubular atrophy characteristic for chronically obstructed kidney. Chronic tubulointerstitial nephritis can be a consequence of vasculitis but no features of active or healed vasculitis were detected. Peritubular fibrosis around excretory tubules might be due to leukemic cell infiltration, which was one of the reasons of nephrectomy decision. No histological confirmation of this hypothesis was found, even considering the 4-year period of remission of this child's leukemia. Leukemia can adversely affect kidneys in several ways. Common causes of leukemia-associated decreased kidney function include direct parenchymal infiltration by leukemic cells, tumor lysis syndrome, thrombotic microangiopathy, radiation injury, and chemotherapy-induced tubular or vascular toxicity. Other causes that should not be forgotten are some prerenal (i.e., volume depletion, heart failure), postrenal (i.e., ureteral obstruction because of lymphadenopathy), and infectious (BK virus) causes [2].

Acute renal side effects of chemotherapy are well characterized and some authors state that patients will sustain renal injury due to the cytostatic therapy with nearly 95 per cent probability [3]. However, it must be taken into consideration that not only cytostatics can play the principal role in renal impairment. The majority of children treated for hemoblastosis are exposed to wide spectrum of other nephrotoxic drugs, in particular some antibiotics [3].

Clinical symptoms of renal injury may manifest as acute renal failure or as different symptoms of chronic renal impairment. There is a paucity of data concerning late nephrotoxicity that may interfere with the child's development and cause permanent morbidity [4]. Therefore, the principal questions are to determine possible acute and chronic nephropathy risks of anticancer therapy and to standardize evaluation of kidney function in children after the end of treatment.

If these parameters (urine microscopy and biochemistry, serum concentrations of urea, creatinine and uric acid,) are monitored in the course of the cytostatic therapy, the acute impairment of the kidneys can be diagnosed early. Most patients experience some spontaneous recovery from acute nephrotoxicity after completing antineoplastic therapy. It is recommended to monitor these patients thoroughly as they represent a risk group for the development of possible irreversible changes of renal function [4]. A question, therefore, appears of whether in the initial phases of cytostatic therapy another biochemical parameters should be monitored, which would signalize milder changes in the glomerular and/or tubular renal functions and what parameters must be monitored to detect late nephrotoxic effects.

Literary data state that patient should be followed up after cessation of therapy with such tests: urinalysis, urinary creatinine and calcium, *β*2-microglobulin, glomerular filtration rate, tubular phosphorus reabsorption, and ultrasonography [5].

The most frequent findings signalizing the renal impairment are proteinuria and impairment of the concentration ability of the kidneys [3]. In case of any abnormality, further detailed tests should be performed and renal scan with DMSA or 99Tc-mercaptoacetyltriglycine (MAG-3) seem to be very predictive for renal damage [5]. In our case, the pathogenesis and the role of hydronephrosis is not completely clear. The diagnosis of intermittent hydronephrosis is suggested by the presence of an accessory vessel as an extrinsic obstacle to urinary flow and several normal renal ultrasound examinations. Classically the main clinical symptom of intermittent hydronephrosis is recurrent abdominal/flank pain, usually accompanied by nausea and vomiting [6, 7]. Other symptoms can be urinary infection, hematuria [7]. Abdominal pain and nausea could have passed without investigation during the course of chemotherapy because of being attributed to the side effects of chemotherapy. At the terminal stage of renal damage with increased diuresis of nonconcentrated urine, hydronephrosis became permanent but did not cause symptoms. In case of intermittent hydronephrosis, surgical indications are difficult to standardize. Important hydronephrosis should be demonstrated on imaging studies (ultrasonography, intravenous urography, and diuretic renography) during an episode of pain [6, 7]. Between obstructive episodes, renal pelvic wall thickening can be seen on ultrasonography [6]. Color Doppler ultrasonography helps to suspect an aberrant renal vessel. It is reported that these children show a greater reduction in differential renal function preoperatively, in contrast to patients without crossing vessel, therefore, recommending an early pyeloplasty [8]. General consensus is to perform nephrectomy if the kidney comprises less than 10 percent of differential function as assessed by a nuclear scan, probably with exceptions in small children and bilateral renal damage. Having only hypothesis of the development of renal damage in our case, we recommend that children with dilatation of renal collecting system and malignant disease should be carefully evaluated and followed.
